# Supplementary material for: Resolving the full spectrum of human genome variation using Linked-Reads
Source: Genome Res. 2019 Apr;29(4):635–45. doi: 10.1101/gr.234443.118 (PMC6442396; doi:10.1101/gr.234443.118)
Supplement: Supplemental Material [file supp_gr.234443.118_Supplemental_longranger-2.2.zip › longranger-2.2.tar.bz2!/lr2.2-public/lib/stan/cmdstan/stan/doc/api/html/namespacemembers_v.html]

Stan: Namespace Members


|  |  |
| --- | --- |
|  | Stan  2.5.0  probability, sampling & optimization |


- Main Page
- Related Pages
- Namespaces
- Classes
- Files

- Namespace List
- Namespace Members

- All
- Functions
- Variables
- Typedefs
- Enumerations
- Enumerator

- a
- b
- c
- d
- e
- f
- g
- h
- i
- j
- l
- m
- n
- o
- p
- q
- r
- s
- t
- u
- v
- w


All Classes Namespaces Files Functions Variables Typedefs Enumerations Enumerator Friends Macros Pages

Here is a list of all namespace members with links to the namespace documentation for each member:

### - v -

- validate\_allow\_sample\_f
  : stan::gm
- validate\_assignment\_f
  : stan::gm
- validate\_declarations\_f
  : stan::gm
- validate\_expr\_type3\_f
  : stan::gm
- validate\_int\_expr2\_f
  : stan::gm
- validate\_int\_expr3\_f
  : stan::gm
- validate\_integrate\_ode\_f
  : stan::gm
- validate\_non\_negative\_index()
  : stan::math
- validate\_non\_void\_arg\_f
  : stan::gm
- validate\_non\_void\_expression\_f
  : stan::gm
- validate\_return\_allowed\_f
  : stan::gm
- validate\_return\_type\_f
  : stan::gm
- validate\_sample\_f
  : stan::gm
- validate\_void\_return\_allowed\_f
  : stan::gm
- value\_of()
  : stan::agrad
  , stan::math
- value\_of< double >()
  : stan::math
- var\_alloc\_stack\_
  : stan::agrad
- var\_nochain\_stack\_
  : stan::agrad
- var\_origin
  : stan::gm
- var\_stack\_
  : stan::agrad
- variance()
  : stan::agrad
  , stan::math
- vector\_d
  : stan::math
  , stan::optimization
- vector\_fd
  : stan::agrad
- vector\_ffd
  : stan::agrad
- vector\_ffv
  : stan::agrad
- vector\_fv
  : stan::agrad
- VECTOR\_T
  : stan::gm
- vector\_v
  : stan::agrad
- void\_function\_argument\_origin
  : stan::gm
- void\_function\_argument\_origin\_lp
  : stan::gm
- void\_function\_argument\_origin\_rng
  : stan::gm
- VOID\_T
  : stan::gm
- von\_mises\_log()
  : stan::prob
- von\_mises\_rng()
  : stan::prob


---

[ Stan Home Page ]

*© 2011–2014,
Stan Development Team.*
